# Identification of a apoptosis-related LncRNA signature to improve prognosis prediction and immunotherapy response in lung adenocarcinoma patients

**DOI:** 10.3389/fgene.2022.946939

**Published:** 2022-09-12

**Authors:** Ting Luo, Shiqun Yu, Jin Ouyang, Fanfan Zeng, Liyun Gao, Shaoxin Huang, Xin Wang

**Affiliations:** ^1^ Jiangxi Provincial Key Laboratory of Preventive Medicine, Nanchang University, Nanchang, Jiangxi, China; ^2^ School of Medicine, Jiujiang University, Jiujiang, Jiangxi, China

**Keywords:** lung adenocarcinoma, apoptosis, long non-coding RNA, prognostic, signature, immunotherapy

## Abstract

Apoptosis is closely associated with the development of various cancers, including lung adenocarcinoma (LUAD). However, the prognostic value of apoptosis-related lncRNAs (ApoRLs) in LUAD has not been fully elucidated. In the present study, we screened 2, 960 ApoRLs by constructing a co-expression network of mRNAs-lncRNAs associated with apoptosis, and identified 421 ApoRLs that were differentially expressed between LUAD samples and normal lung samples. Sixteen differentially expressed apoptosis-related lncRNAs (DE-ApoRLs) with prognostic relevance to LUAD patients were screened using univariate Cox regression analysis. An apoptosis-related lncRNA signature (ApoRLSig ) containing 10 ApoRLs was constructed by applying the Least Absolute Shrinkage and Selection Operator (LASSO) Cox regression method, and all LUAD patients in the TCGA cohort were divided into high or low risk groups. Moreover, patients in the high-risk group had a worse prognosis (*p* < 0.05). When analyzed in conjunction with clinical features, we found ApoRLSig to be an independent predictor of LUAD patients and established a prognostic nomogram combining ApoRLSig and clinical features. Gene set enrichment analysis (GSEA) revealed that ApoRLSig is involved in many malignancy-associated immunomodulatory pathways. In addition, there were significant differences in the immune microenvironment and immune cells between the high-risk and low-risk groups. Further analysis revealed that the expression levels of most immune checkpoint genes (ICGs) were higher in the high-risk group, which suggested that the immunotherapy effect was better in the high-risk group than in the low-risk group. And we found that the high-risk group was also better than the low-risk group in terms of chemotherapy effect. In conclusion, we successfully constructed an ApoRLSig which could predict the prognosis of LUAD patients and provide a novel strategy for the antitumor treatment of LUAD patients.

## Introduction

Lung cancer still has the highest mortality rate in the cancer spectrum worldwide, with a 5-years survival rate of only 10%–20% (Sung et al., 2021). Lung adenocarcinoma (LUAD) accounts for approximately 40%–50% of all lung cancer cases (Bray et al., 2018). Although molecular targeted therapies and immunotherapies have been developed for LUAD, long-term survival remains suboptimal for most patients (Saito et al., 2018). Therefore, it remains urgent to identify new and effective prognostic biomarkers to improve the low survival rate of patients with LUAD.

Apoptosis is one of the most common and well-studied forms of programmed cell death (Fuchs and Steller, 2015), the initiation of which depends on the activation of a series of Caspase proteases that subsequently induce extensive cleavage of hundreds of substrates and rapid cell death (D'Arcy, 2019). Apoptosis has a dual role in cancer, on the one hand, it can inhibit tumor development by deleting malignant or pre-malignant cells; on the other hand, it can promote tumor development by stimulating reparative and regenerative responses in the tumor microenvironment (Morana et al., 2022). Moreover, apoptosis plays an important role in the development and progression of non-small cell lung cancer, and targeting apoptosis may be a new and effective treatment for lung cancer (Liu et al., 2017).

Long non-coding RNAs (lncRNAs), which typically exceed 200 nucleotides in size and are transcribed by RNA polymerase II, have an important regulatory role in the induction of apoptosis (Ghafouri-Fard et al., 2021). Many studies have demonstrated that lncRNAs are key regulators involved in the progression of human cancer including lung cancer. Different lncRNAs can modulate the sensitivity of chemotherapy, radiotherapy and egfr-targeted therapy through distinct mechanisms (Chen Y. et al., 2021b). In recent years, several studies have constructed a series of prognostic lncRNA signatures in LUAD to improve patient prognosis by exploring lncRNAs associated with ferroptosis (Lu et al., 2021), pyroptosis (Song et al., 2021), autophagy (Chen et al., 2021), necroptosis (Lu et al., 2022), and immunity (Wu G. et al., 2021). Whereas, the apoptosis-related lncRNA signature (ApoRLSig) and its relationship with prognosis have not been systematically evaluated in LUAD.

In this study, an ApoRLSig was constructed based on The Cancer Genome Atlas (TCGA) database, and the relevance of apoptosis-related lncRNAs (ApoRLs) to the prognosis of patients with LUAD was systematically assessed. Then, we analyzed the relationship between ApoRLs and clinicopathological characteristics of LUAD patients, and established a nomogram to individually predict patient’s survival. In addition, the relationship between risk score and tumor immune microenvironment, immune checkpoint genes (ICGs), and chemotherapy sensitivity was further evaluated. The results of this study may help to improve individualized treatment effectiveness and prognostic assessment of patients with LUAD.

## Materials and methods

### Data acquisition and processing

RNA sequencing (RNA-seq) data and corresponding clinical survival information for LUAD samples from TCGA database were downloaded via the UCSC xena website (https://xenabrowser.net/datapages/). There were 510 tumor samples and 58 normal samples in the TCGA-LUAD dataset. To reduce statistical bias in the analysis, patients with missing overall survival (OS) or short survival (<30 days) were excluded, and 487 patients were finally included in the study (Supplementary Table S1). A total of 326 patients with complete clinicopathological data were included in the subsequent analysis (Supplementary Table S2).

### Apoptosis-related gene detection

A total of 136 apoptosis-related genes were collected by searching the Kyoto Encyclopedia of Genes and Genomes (KEGG) pathway database (https://www.kegg.jp/kegg/pathway.html) with the keyword “Apoptosis.” Eventually, 134 apoptosis-related genes were retrieved from the mRNA expression profile of TCGA-LUAD (Supplementary Table S3).

### Screening of apoptosis-related lncRNAs

Pearson correlation analysis was performed to identify potential lncRNAs associated with apoptosis-related genes. The apoptosis-related mRNA-lncRNA co-expression network was constructed using |Pearson correlation coefficient|>0.4 and *p* < 0.001 as thresholds. A total of 2, 960 ApoRLs were identified. The co-expression network was visualized using Cytoscape 3.8.2. Using the R package “ggalluvial” to draw sankey diagrams. Differentially expressed ApoRLs (DE-ApoRLs) between tumor and normal samples were identified by the “DESeq2” package (Love et al., 2014). |log2FC|>2 and FDR<0.05 were considered to be significant.

### Construction of apoptosis-related lncRNA prognostic model in lung adenocarcinoma

Univariate Cox analysis of OS was performed to identify DE-ApoRLs with prognostic value (*p* < 0.001). Then, using the R package “glmnet,” the least absolute shrinkage and selection operator (LASSO) Cox regression was performed to screen for key DE-ApoRLs. Risk scores of patients were calculated based on the expression levels of lncRNAs and the corresponding lasso coefficients. The risk score is calculated by the formula: risk score = ∑exp(i)×coef(i). Using the median risk score as the cut-off point, patients were divided into a low-risk group and a high-risk group. Survival analysis was performed to compare the OS of the high-risk and low-risk groups by using the R packages “survivor” and “survminer.” Using the R package “timeROC,” time-dependent receiver operating characteristic analysis and the area under the curve (AUC) were performed to assess the predictive power of the model. Principal component analysis (PCA) was performed to evaluate the distribution of patients with different risk scores, and PCA plots were generated by the “scatterplot3D” package of R. In addition, the distribution of patient survival status was evaluated based on risk score levels.

### Predictive nomogram construction

The Wilcoxon test was used to explore the potential relationship between the risk score and multiple clinical characteristics (age, sex, stage, TNM stage). *p* < 0.05 was considered to be significant. Then, univariate and multivariate Cox regression analyses were performed on the independent prognostic factors, and the results were visualized using the R package “forestplot.” Subsequently, independent risk factors with clinical prognostic significance were integrated, and a nomogram was constructed to predict 1-, 3-, and 5-years survival in LUAD patients by using the R package “rms.” Finally, the predictive accuracy of the model was further evaluated by the consistency index, calibration curve, and receiver operating characteristic (ROC) curve.

### Gene set enrichment analysis

Gene set enrichment analysis was performed for genes in the high-risk and low-risk group using the R package “clusterProfiler” and “org.Hs.eg.db.” The c5.go.v7.5.1.entrez.gmt and c2.cp.kegg.v7.5.1.entrez.gmt were selected as predefined gene sets from the Molecular Signature Database (MSigDB; https://www.gsea-msigdb.org/gsea/msigdb/index.jsp). Biological processes and pathways that were significantly enriched were screened according to the criteria of NOM *p* < 0.05 and FDR<0.25.

### Immune infiltration and chemotherapeutic drug sensitivity analysis

The immune, stromal and estimete score for each patient were calculated by the R “estimate” package. The level of immune cell infiltration was quantified for each patient using CIBERSORT (https://cibersort.stanford.edu/). A heat map of the correlation between lncRNAs and immune cell infiltration was drawn by the R package “corrplot.” The proportions of 22 immune cells in the high- and low-risk groups were compared and the results were visualized using the R package “vioplot.” In addition, the single-sample gene set enrichment analysis (ssGSEA) in the “GSVA” package was used to quantify the relative infiltration of 28 immune cell types in the tumor microenvironment (Barbie et al., 2009). The set of characteristic genes for each immune cell type was obtained from a publication (Jia et al., 2018). In the ssGSEA analysis, the relative abundance of each immune cell type was represented by an enrichment score. Seventy-nine ICGs were obtained from the literature (Hu et al., 2021), 78 of which were expressed in the TCGA-LUAD dataset, and the relationship between the risk score and expression levels of ICGs was assessed. The IC50 values of common antitumor drugs used in the treatment of LUAD, such as cisplatin, etoposide, docetaxel, gefitinib, erlotinib, gemcitabine, and paclitaxel, were compared between two groups using the R packages “pRRophetic” and “ggplot2”.

### Statistical analysis

All calculations and statistical analyses for this study were performed in R (version 4.1.3) (https://www.r-project.org/). Survival analysis was performed using the Kaplan-Meier method. The Wilcoxon signed-rank test was used to compare the differences between groups. Spearman or Pearson correlation coefficients were used to evaluate the relationships among lncRNA expression, estimate scores, and immune infiltration. *p* < 0.05 was considered a significant difference.

## Results

### Identification of apoptosis-related lncRNAs with prognostic value in lung adenocarcinoma

**FIGURE 1:**
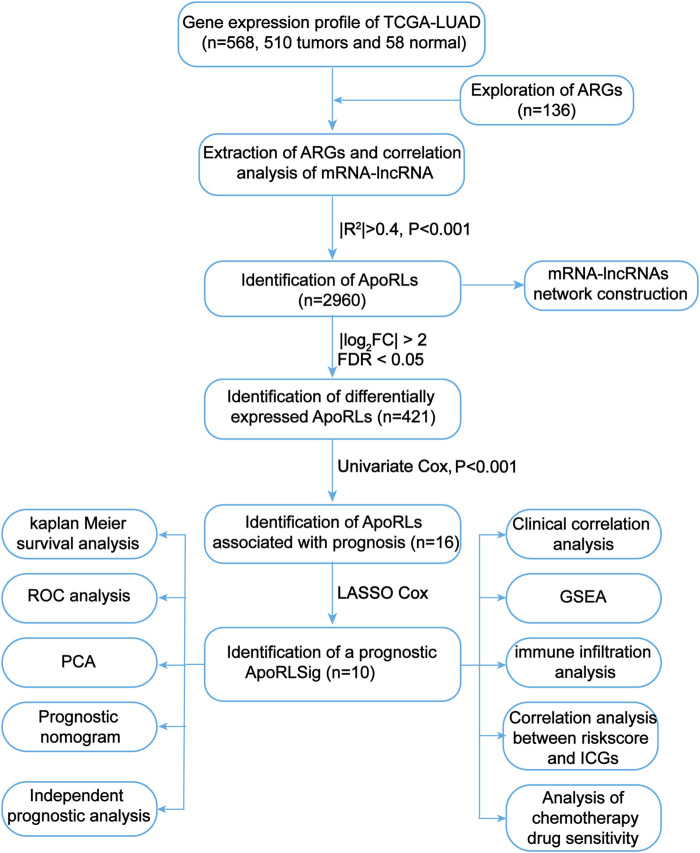
Flowchart of the present study. TCGA, The Cancer Genome Atlas; LUAD, lung adenocarcinoma; ARGs, apoptosis-related genes; ApoRLs, apoptosis-related lncRNAs; ApoRLSig, apoptosis-related lncRNA signature; ICGs, immune checkpoint genes.

**FIGURE 2:**
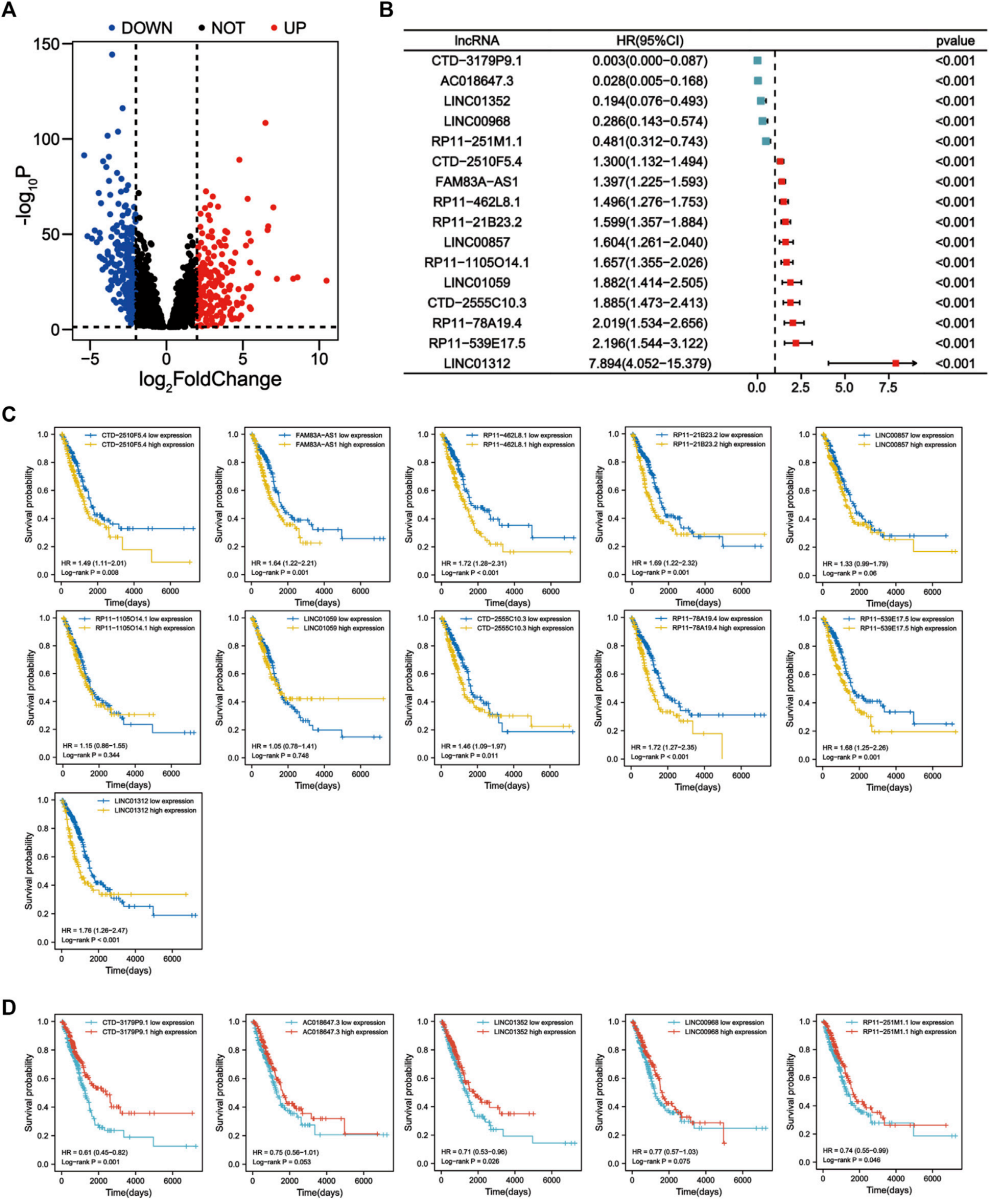
Identification of prognostic apoptosis-related lncRNAs in LUAD patients. **(A)** The differentially expressed apoptosis-related lncRNAs were shown in the volcano plot. **(B)** Forest plot showing the HR (95% CI) and *p* values of lncRNAs screened by univariate Cox regression analysis (all *p* < 0.001). **(C)** Kaplan–Meier survival curves for eleven unfavorable prognostic apoptosis-related lncRNAs. **(D)** Kaplan-Meier survival curves for five apoptosis-related lncRNAs with a good prognosis.

We first screened 136 apoptosis-related genes (mRNAs), of which 134 genes had expression data in the TCGA-LUAD dataset (Supplementary Table S3). The workflow of this study is shown in Figure 1. Peasron correlation analysis identified 2, 960 ApoRLs (|*R* ^2^|>0.4, *p* < 0.001). Then, differential analysis of tumor and normal samples identified 421 DE-ApoRLs (|log_2_FC|>2, *p* < 0.05, Figure 2A; Supplementary Figure S1A). Next, 16 lncRNAs whose expression levels correlated with patient prognosis were screened by univariate Cox regression, suggesting their prognostic value for LUAD (*p* < 0.001, Figure 2B; Supplementary Table S4). Eleven lncRNAs were poor prognostic factors (HR > 1, Figure 2C) and five lncRNAs were favorable prognostic factors (HR < 1, Figure 2D).

### Construction of a prognostic apoptosis-related lncRNA signature

**FIGURE 3:**
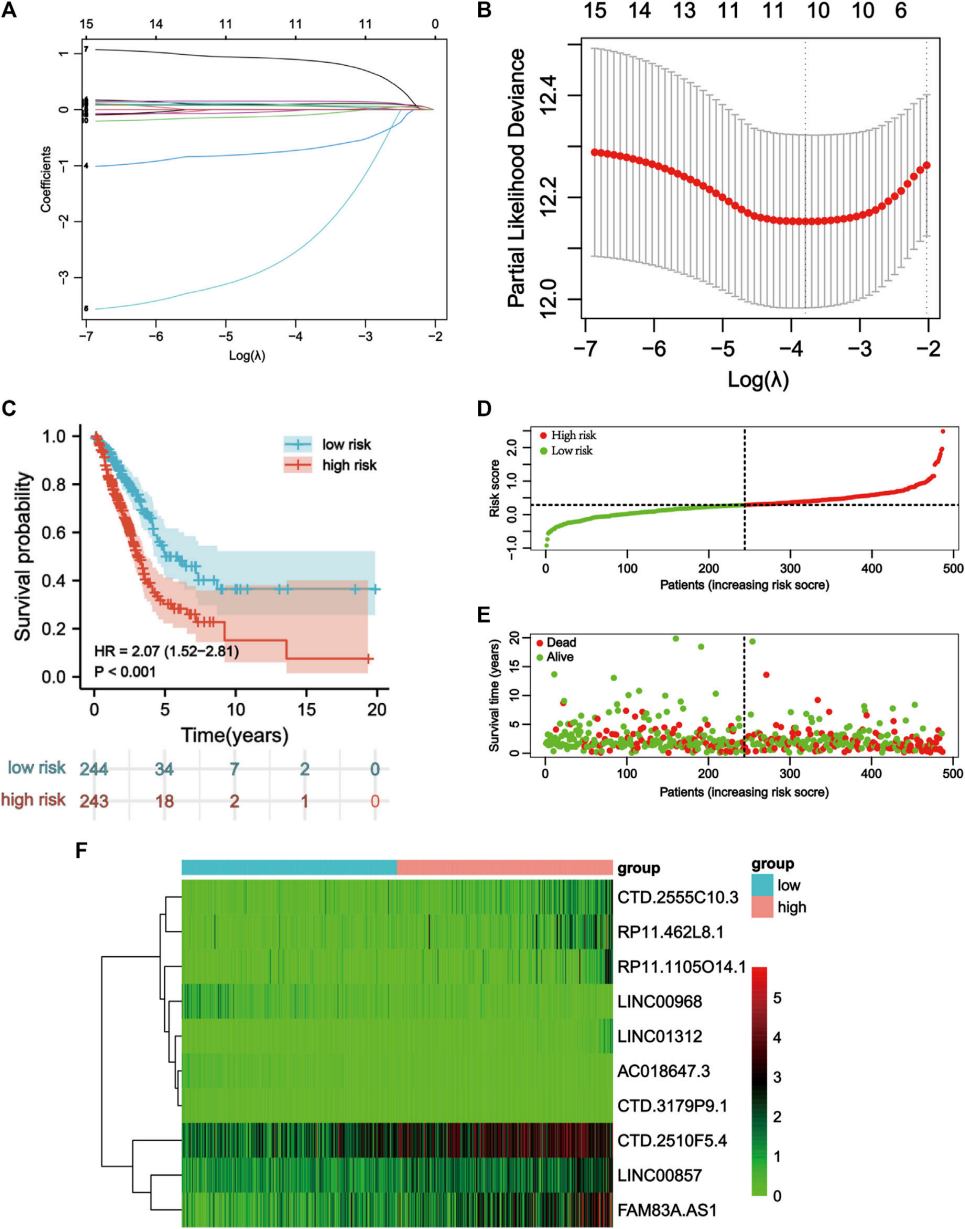
Construction of the apoptosis-related lncRNA signature. **(A)** Lasso coefficients profiles of the 16 apoptosis-related lncRNAs. **(B)** Lasso regression analysis obtained 10 prognostic apoptosis-related lncRNAs. **(C)** Kaplan–Meier curves for OS in the high-risk and low-risk groups stratifified by ApoRLSig (*p* < 0.001). **(D)** Risk curve based on the risk score for each sample, where red indicates a high risk and green indicates a low risk. **(E)** Scatterplot based on the survival status of each sample. Red and green dots indicate death and survival, respectively. **(F)** The heatmap shows the distribution of 10 apoptosis-related lncRNAs in the high-risk and low-risk groups.

**FIGURE 4:**
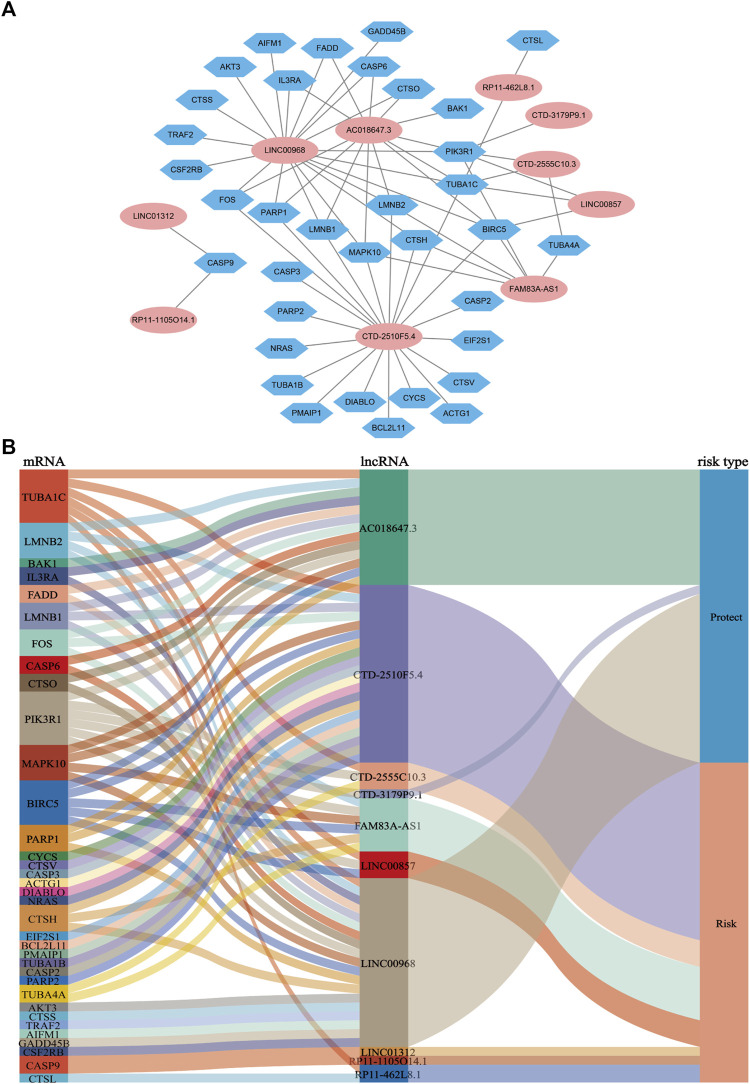
Coexpression network and Sankey diagram of prognostic apoptosis-related lncRNAs. **(A)** A co-expression network of apoptosis-related lncRNAs and mRNAs was constructed. Pink ellipses indicate prognostic AR-lncRNAs, and blue hexagons indicate apoptosis-related mRNAs. The levels of the 10 apoptosis-related lncRNAs were associated with the levels of 35 apoptosis-related mRNAs. **(B)** Sankey diagram showing the associations between prognostic apoptosis-related lncRNAs, mRNAs, and risk type.

LASSO Cox regression analysis identified 10 ApoRLs (RP11.1105O14.1, CTD.2510F5.4, AC018647.3, CTD.3179P9.1, CTD.2555C10.3, LINC01312, LINC00968, RP11.462L8.1, LINC00857, FAM83A.AS1) , and established a prognostic ApoRLSig (Figures 3A,B). The correlations of these 10 lncRNAs with apoptosis genes are shown in Figure 4A. Among them, seven lncRNAs (RP11.1105O14.1, CTD.2510F5.4, CTD.2555C10.3, LINC01312, RP11.462L8.1, LINC00857, FAM83A.AS1) were significant adverse prognostic factors, while the remaining lncRNAs (AC018647.3, CTD.3179P9.1, LINC00968) were favorable prognostic factors for OS (Figure 4B). The risk score was calculated as follows: risk score = (0.1072 × RP11.1105O14.1 expression level) + (0.0555 × CTD.2510F5.4 expression level) + (−0.6456 × AC018647.3 expression level) + (−2.0068 × CTD.3179P9.1 expression level) + (0.1503 × CTD.2555C10.3 expression level) + (0.8414 × LINC01312 expression level) + (−0.0628 × LINC00968 expression level) + (0.0605 × RP11.462L8.1 expression level) + (0.0702 × LINC00857 expression level) + (0.0708 × FAM83A.AS1 expression level). We calculated the risk score for each patient according to the formula, and divided patients into a high-risk group (n = 243) and a low-risk group (*n* = 244) using the median risk score as the threshold. Kaplan-Meier curves showed a significant difference in OS between the high-risk and low-risk groups of LUAD patients (*p* < 0.001, Figure 3C), indicating that the newly developed signature is effective in predicting survival. Meanwhile, the risk curve, scatter plot based on survival status and heat maps of expression distribution for these 10 lncRNAs are shown in Figures 3D–F.

### Evaluation of ApoRLSig as an independent prognostic factor for lung adenocarcinoma

**FIGURE 5:**
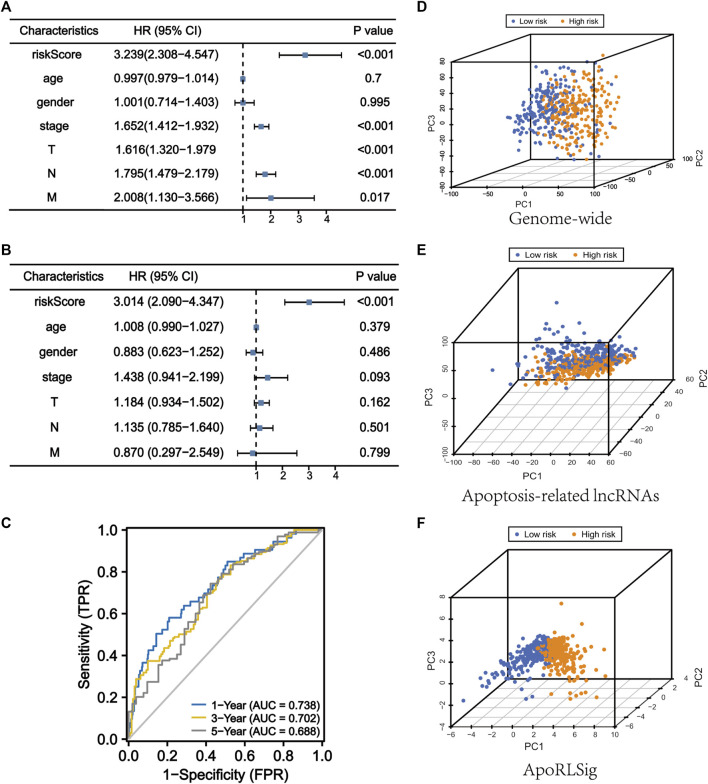
ApoRLSig is an independent prognostic factor for overall survival. Univariate **(A)** and multivariate **(B)** Cox regression analysis of the relationship between clinical characteristics (including FerRLSig) and OS. **(C)** Time-dependent ROC curves of OS at 1, 3, and 5 years. Principal component analysis (PCA) of low-risk and high-risk groups based on the **(D)** genome-wide, **(E)** apoptosis-related lncRNAs, and **(F)** the ApoRLSig including 10 apoptosis-related lncRNAs. Patients with high risk scores are indicated in orange, and those with low risk scores areindicated in blue. T, tumor stage; N, lymph node metastasis stage; M, distant metastasis stage.

We performed univariate and multivariate Cox regression analyses to determine whether ApoRLSig is an independent prognostic model for OS in LUAD patients. The HRs (95% CI) for the risk score in univariate and multivariate Cox regression analyses were 3.239 (2.308–4.547) (*p* < 0.001, Figure 5A) and 3.014 (2.090–4.347) (*p* < 0.001, Figure 5B), indicating that ApoRLSig is an independent prognostic indicator. In addition, the predictive accuracy of the model was assessed by time-dependent receiver operating characteristic analysis at 1, 3, and 5 years, with AUC values of 0.738, 0.702, and 0.688, respectively (Figure 5C). Then, we compared the low-risk and high-risk groups based on genome-wide, ApoRLs, and the risk model using PCA. As shown in Figures 5D,E, genome-wide or ApoRLs could not effectively distinguish between high-risk and low-risk groups, while ApoRLSig could clearly distinguish between high-risk and low-risk patients, further supporting the accuracy of the model (Figure 5F). The above results illustrated that ApoRLSig is an important independent prognostic risk factor for patients with LUAD.

### Correlations between the risk score and clinicopathological factors

**FIGURE 6:**
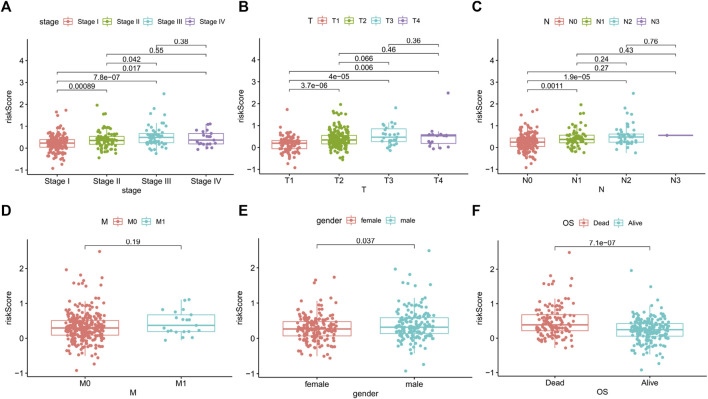
ApoRLSig was associated with the clinicopathological factors of patients with LUAD. Correlation analysis between risk score and Stage **(A)**, T **(B)**, N **(C)**, M **(D)**, Gender **(E)**, survival outcome **(F)**. T, tumor stage; N, lymph node metastasis stage; M, distant metastasis stage.

To further assess the role of ApoRLSig in the development of LUAD, we evaluated the correlations between the risk score and clinicopathological factors. As shown in Figure 6 and Supplementary Table S5, there was a significant correlation between the risk score and pathological stage (*p* < 0.01), especially for stages II-IV, which were significantly higher than stage I (Figure 6A, *p* < 0.05). The signature correlated with tumor stage (Figure 6B, *p* < 0.05), and patients with lymph node metastases had significantly higher risk scores than those without lymph node metastases (Figure 6C, *p* < 0.01). In addition, there was a correlation between the signature and gender (Figure 6E, *p* < 0.05). Figure 6F illustrated that patients with high risk scores had a significantly poorer prognosis in terms of survival status than patients with low-risk scores. These results suggested that ApoRLSig is closely associated with the progression and prognosis of LUAD.

### Construction of a predictive nomogram

**FIGURE 7:**
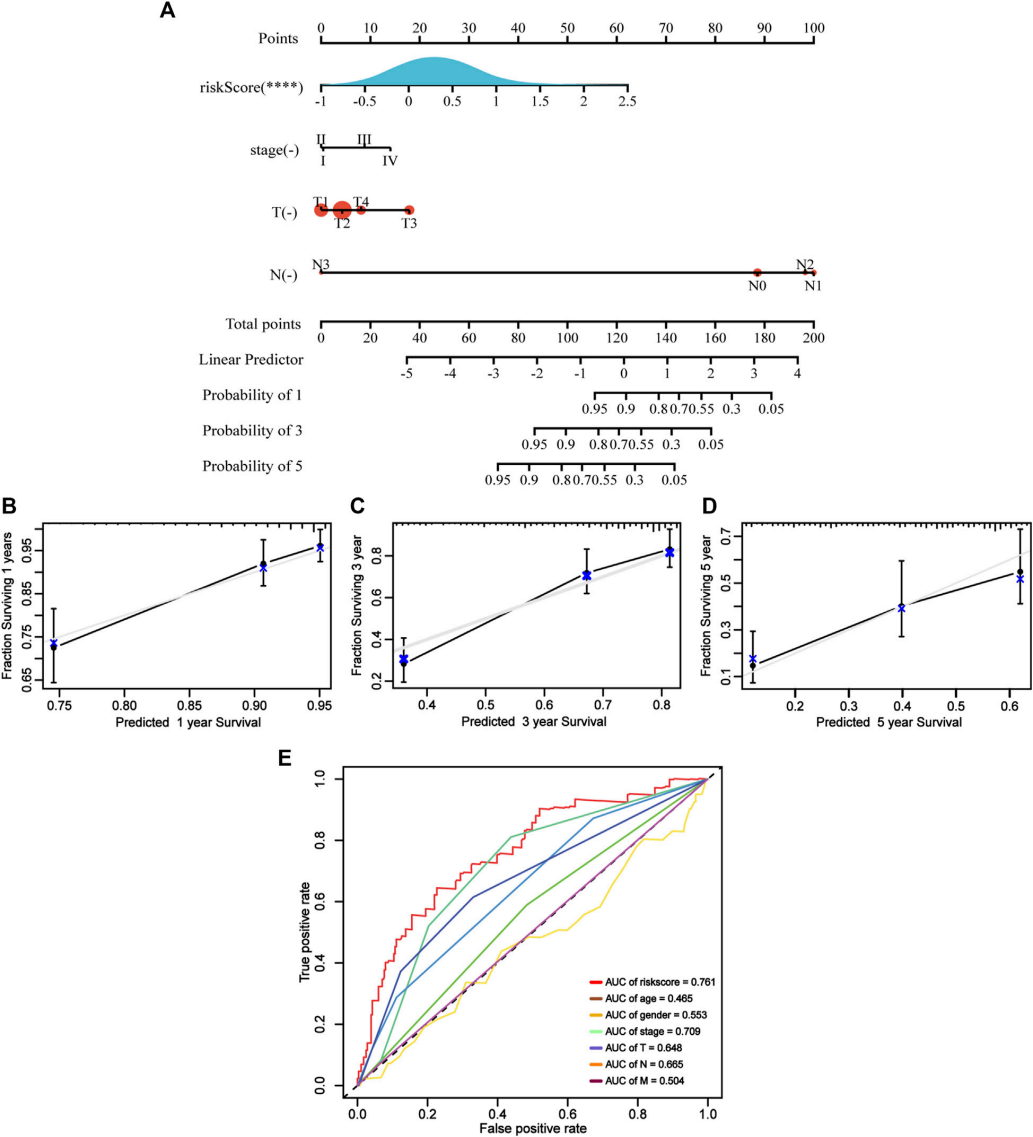
Clinical predictive nomogram construction and evaluation. **(A)** A clinical predictive nomogram based on the clinicopathological factors and risk score.The calibration curves of the nomogram in predicting 1-year **(B)**, 3-years **(C)**, and 5-years **(D)** survival of LUAD patients. **(E)** Time-dependent ROC curve analyses for predicting OS at 1 year by risk score age, sex, stage, T (tumor size), N (lymph node metastasis and M (distant metastasis).

Using ApoRLSig in combination with other clinicopathological factors (stage, T, and N), we constructed a clinically applicable nomogram to estimate the probability of survival at 1, 3 and 5 years for patients with LUAD (Figure 7A). The consistency index of the model was: 0.73 (95% CI: 0.68–0.78, *p* < 0.001) and its 1-, 3-, and 5-years calibration curves indicated that the mortality rates estimated by the nomogram were close to the actual mortality rates (Figures 7B–D). In the time-dependent ROC curve for 1-year OS, the AUC value of ApoRLSig was 0.761, which was significantly higher than other clinical features, further supporting the predictive ability of ApoRLSig for survival in patients with LUAD (Figure 7E).

### Identification of ApoRLSig-related biological pathways

**FIGURE 8:**
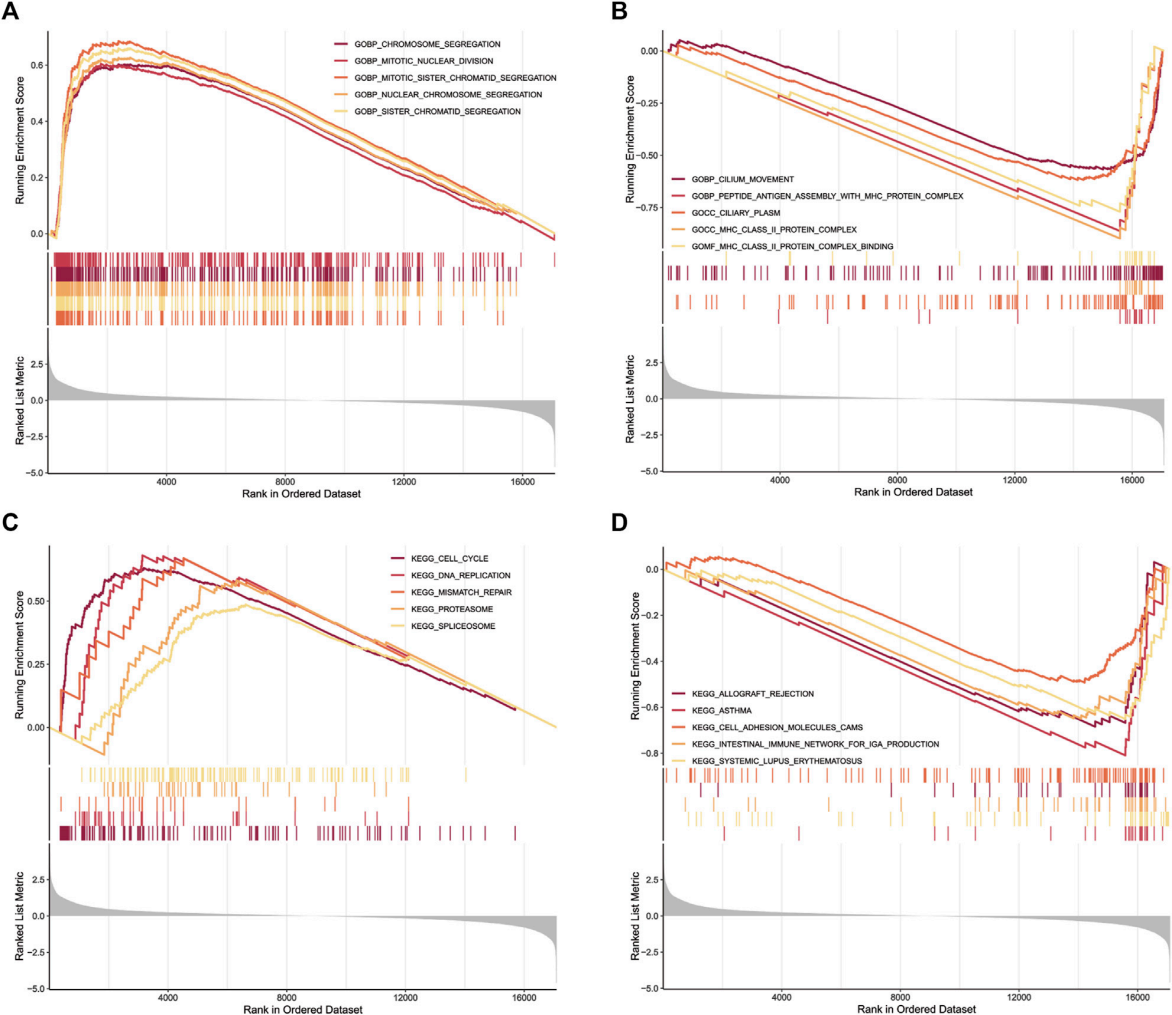
GSEA for samples with high risk scores and low risk scores. **(A)** Enriched gene sets in C5 collection, the Go gene sets, by patients with high risk scores. Only gene sets both with NOM *p* < 0.05 and FDR q < 0.25 were considered signifificant. Only five top gene sets are shown in the plot. **(B)** The enriched gene sets in C5 collection by patients with low risk scores. **(C)** Enriched gene sets in C2 collection, the KEGG gene sets, by patients with high risk scores. **(D)** The enriched gene sets in C2 collection by patients with low risk scores.

GO functional annotation and KEGG pathway enrichment were performed using Gene set enrichment analysis. GO functional annotation results (Supplementary Table S6) showed that chromosome segregation (NES = 2.50, *p* = 0.000), mitotic nuclear division (NES = 2.48, *p* = 0.000), mitotic sister chromatid segregation (NES = 2.63, *p* = 0.000), nuclear chromosome segregation (NES = 2.54, *p* = 0.000) and sister chromatid segregation (NES = 2.58, *p* = 0.000) were enriched in LUAD patients with high risk scores (Figure 8A). In contrast, cilium movement (NES = −2.19, *p* = 0.000), peptide antigen assembly with MHC protein complex (NES = −2.27,*p* = 0.000), ciliary plasm (NES = −2.26, *p* = 0.000), MHC class II protein complex (NES = −2.23, *p* = 0.000) and MHC class II protein complex binding (NES = −2.18, *p* = 0.0002) were enriched in patients with low risk scores (Figure 8B). In addition, 19 KEGG pathways were enriched (Supplementary Table S7). Cell cycle (NES = 2.35, *p* = 0.000), DNA replication (NES = 2.07, *p* = 0.001), mismatch repair (NES = 1.84, *p* = 0.031), proteasome (NES = 1.82, *p* = 0.014) and splicesome (NES = 1.81, *p* = 0.001) signaling pathways were enriched in the high-risk group (Figure 8C). Meanwhile, Allograft rejection (NES = −2.05, *p* = 0.001), asthma (NES = −2.26, *p* = 0.000), cell adhesion molecules CAMs (NES = -1.83, *p* = 0.001), intestinal immune network for IgA production (NES = −2.06, *p* = 0.001) and systemic lupus erythematosus (NES = -2.12, *p* = 0.000) signaling pathways were enriched in the low-risk group (Figure 8D). We found that multiple of these pathways are immune response-related pathways. The results indicated that the lncRNAs signature may be related to the tumor immune microenvironment.

### Correlation of the risk score with tumor immune microenvironment

**FIGURE 9:**
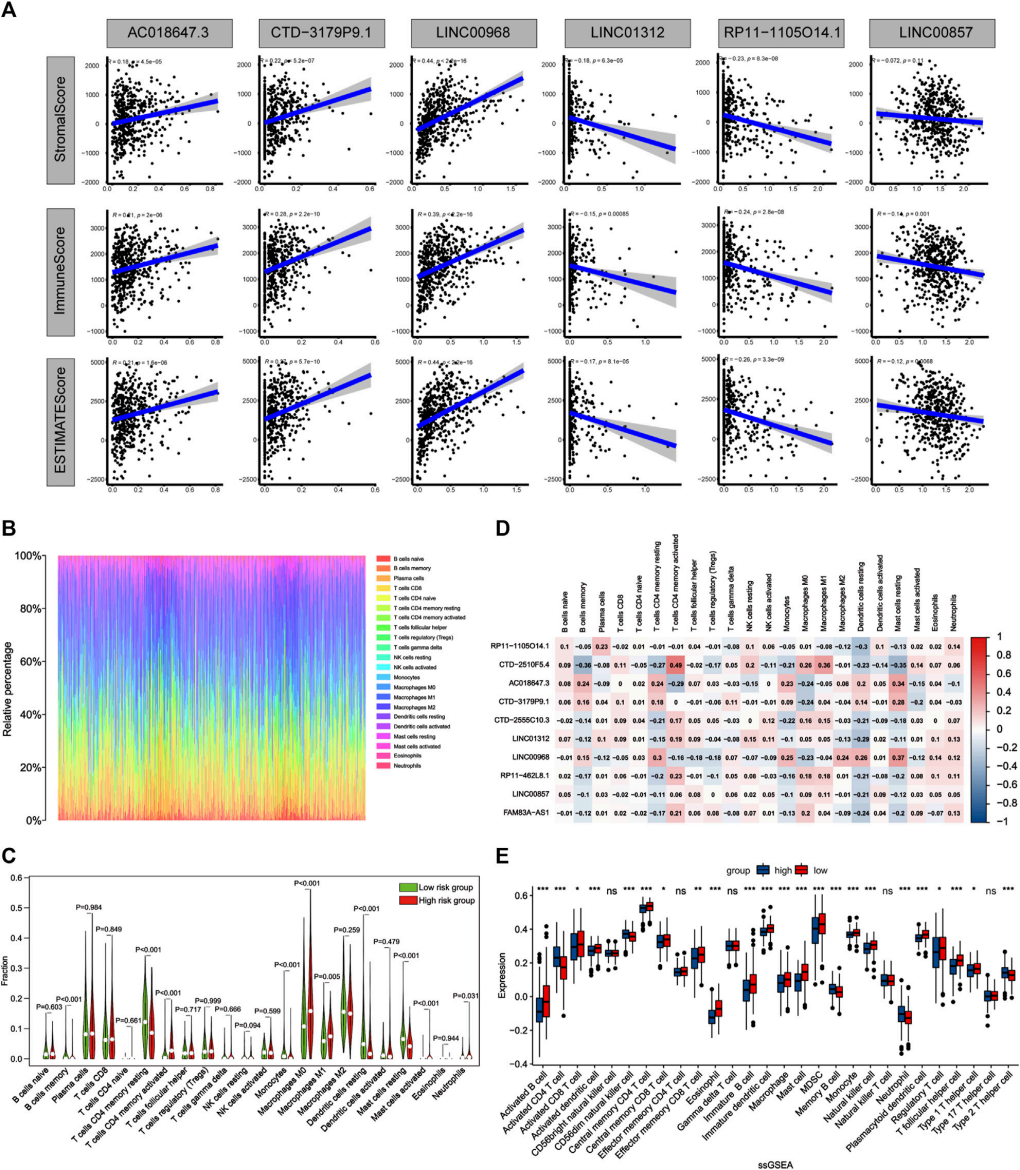
Comparison of the immune microenvironment of LUAD patients between the high- and low-risk groups. **(A)** Correlation matrices between six lncRNAs expression and stromal score, immune score, and estimate score. **(B)** Barplot shows the proportion of 22 types of TICs in LUAD samples. The column names of the plot were sample ID. **(C)** Violin plot showed the ratio of 22 immune cell types between the low-risk and high-risk groups, and Wilcoxon signed-rank test was used for significance test. Red indicates the high-risk group and green indicates the low-risk group. **(D)** Heatmap showing the correlation between 22 TICs and 10 lncRNAs. **(E)** The single sample gene set enrichment analysis (ssGSEA) algorithm compares the expression of 28 immune cells between patients in high and low risk groups. *p < 0.05; **p < 0.01; ***p < 0.001.

To further assess the correlations between the risk score and tumor microenvironment, we quantified the level of tumor immune cells infiltration in both groups of patients using ESTIMATE, CIBERSORT and ssGSEA algorithms. The results showed that three ApoRLs were positively correlated with stromal, immune and estimate score, including AC018647.3, CTD-3179P9.1 and LINC00968, while LINC01312, RP11-1105O14.1 and LINC00857 were negatively correlated with them (*p* < 0.001, Figure 9A; Supplementary Figure S1B). Differences in infiltration of 22 immune cell types in patients with LUAD in TCGA are shown in Figure 9B, reflecting the intrinsic characteristics of individual differences. The high-risk group of LUAD patients had a higher proportion of T cells CD4 memory activated (*p* < 0.001), Macrophages M0 (*p* < 0.001), Mast cells activated (*p* < 0.001) and Neutrophils (*p* = 0.031). In contrast, B cells memory (*p* < 0.001), T cells CD4 memory resting (*p* < 0.001), Monocytes (*p* < 0.001), Macrophages M1 (*p* = 0.005), Dendritic cells resting (*p* < 0.001) and Mast cells resting (*p* < 0.001) were negatively associated with risk score (Figure 9C). Furthermore, we analyzed the correlations between 10 ApoRLs and 22 immune cells (Figure 9D). Correlation analysis of immune cell subsets based on ssGSEA showed more immune cell infiltration in the low-risk group, including B cells, central memory CD4^+^ T cells, dendritic cells, natural killer cells, Eosinophi, Macrophage , Mast cells, MDSC , Monocyte , CD8^+^ T cells, T follicular helper cells, Regulatory T cells and Type 1 T helper cell (*p* < 0.001, Supplementary Table S8; Figure 9E). In contrast, only memory B cells, Activated CD4^+^ T cells, CD56dim natural killer cells, neutrophils and Type 2 T helper cells infiltrated in the high-risk group. The results suggested that our signature is not only a prognostic marker but also reflects the level of immune cell infiltration.

### Differences in response to immunotherapy and chemotherapy between the high-risk and low-risk groups

**FIGURE 10:**
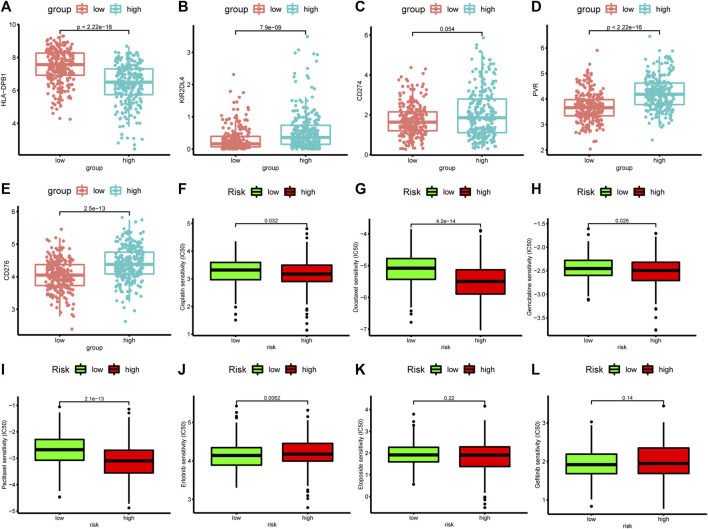
Correlation analysis between risk score, ICGs, and chemotherapeutics sensitivity. The differential expression of five immune checkpoint genes, **(A)** HLA-DPB1, **(B)** KIR2DL4, **(C)** CD27, **(D)** PVR, and **(E)** CD276, between the high-risk group and the low-risk group. Half-maximal inhibitory concentration (IC50) values for seven common antineoplastic drugs, **(F)** cisplatin, **(G)** docetaxel, **(H)** gemcitabine, **(I)** paclitaxel, **(J)** erlotinib, **(K)** etoposide, and **(L)** gefitinib, between the high-risk and low-risk groups.

The expression levels of ICGs may be predictive biomarkers for immune checkpoint blockade therapy. We investigated the relationship between the expression of 78 ICGs and two groups. The results showed that 19 ICGs were expressed at higher levels in the low-risk group, including BTNL9, HLA-DRB5, HLA-DPB1, HLA-DOA, HLA-DQB1, CD40LG, HLA-DRB1, HLA-DRA, HLA-DPA1, HLA-DMA, HLA-DQA1, HLA-DMB, CD96,BTLA, HLA-DOB, CD48, TNFSF15, CD200R1, CD28, while the other 41 ICGs were highly expressed in the high-risk group (Supplementary Table S9). The first 10 ICGs were shown in Figures 10A–E and Supplemenatry Figures S1C–G. These results demonstated that ApoRLSig could be a candidate biomarker for immunotherapy in patients with LUAD. In addition, the results of the correlation analysis between risk score and the sensitivity of chemotherapeutic agents to LUAD were shown in Figures 10F–L. Patients with high risk scores were highly sensitive to cisplatin (*p* = 0.032), docetaxel (*p* < 0.001), gemcitabine (*p* = 0.026) and paclitaxel (*p* < 0.001), while patients with low risk scores were only sensitive to erlotinib (*p* = 0.006). There was no significant difference in the sensitivity of etoposide and gefitinib between the two groups (*p* > 0.05). The results indicate that ApoRLSig is a potential predictor of chemotherapy sensitivity.

## Discussion

Lung cancer is the leading cause of cancer deaths worldwide. LUAD is one of the most common histological types of lung cancer (Carrillo-Perez et al., 2021). In recent years, chemotherapy and molecular targeted therapy can prolong the overall survival of patients with LUAD, and the emergence of immunotherapy also brings a promising future to LUAD treatment (Lou et al., 2020; Deshpand et al., 2022). However, the prognosis for patients with LUAD remains poor due to late diagnosis and the emergence of drug resistance (Blandin et al., 2017; Li et al., 2020). Hence, there is an urgent need to develop safe and feasible predictive biomarkers that will facilitate accurate and timely personalized treatment of LUAD patients and greatly improve their prognosis.

Apoptosis is a specific programmed cell death process regulated by molecules, and regulating apoptosis can treat a variety of diseases, including cancer (Ketelut-Carneiro and Fitzgerald, 2022). Moreover, the cytotoxic effects of most oncological chemotherapeutic agents are mediated through activation of apoptotic pathways, and apoptosis targeting holds promise as a key strategy for cancer treatment (Johnstone et al., 2002; Singh and Lim, 2022). Increasing evidence showed that lncRNAs can regulate apoptosis through different mechanisms, and their regulatory effects on apoptosis in lung cancer cells have been investigated (Wang et al., 2020; Xiang et al., 2020; Ghafouri-Fard et al., 2021; Ouyang et al., 2021). Whereas, the role of ApoRLs in the prognosis, chemotherapy and immunotherapy of LUAD is not well understood.

In this study, we constructed a prognostic signature using 10 ApoRLs, and the ROC curve demonstrated that this lncRNA signature had moderate predictive performance for OS in LUAD patients. We then evaluated the relationship between the risk score and clinical features of LUAD and constructed a nomogram diagnostic model. Next, we linked the lncRNA signature to the tumor immune microenvironment and found that these ApoRLs play a key role in the regulation of tumor immune infiltration, suggesting that they may be potential targets for tumor immunotherapy. Finally, the correlation between ICGs, chemotherapeutic sensitivity and risk score was analyzed to assess the role of this signature in immune response and chemotherapy effect in LUAD. These results strongly suggested that the lncRNA signature may play an important role in LUAD.

Among the identified lncRNAs, five were closely associated with tumor development, namely CTD.2510F5.4, LINC01312, LINC00857, FAM83A.AS1, and LINC00968. CTD.2510F5.4 was found to be significantly upregulated in cancerous tissues and was strongly associated with poor prognosis in LUAD (Wang et al., 2018). We found that LINC01312 could be used as a prognostic marker to predict survival in LUAD (Li et al., 2018). However, the biological functions of LINC01312 in apoptosis and LUAD have not been systematically analyzed and need to be further investigated. LINC00857 is considered to be an oncogenic lncRNA that promotes proliferation and metastasis of cancer cells in pancreatic (Chen et al., 2022), colorectal (Chang et al., 2021) and breast (Zheng et al., 2020) cancers, and it regulates apoptosis and autophagy (Su et al., 2020). FAM83A.AS1 regulates the proliferation, migration, invasion and epithelial-mesenchymal transition process of LUAD cells by targeting microRNA-141-3p (Huang et al., 2022). Notably, LINC00857 and FAM83A.AS1 are components of the immune-associated lncRNA signature (Mu et al., 2021; You et al., 2021), suggesting a possible strong link between apoptosis and immune regulation in LUAD. LINC00968 is significantly downregulated in LUAD and inhibits tumor proliferation, migration and invasion, and may serve as a prognostic marker and potential therapeutic target for LUAD (Wu C. et al., 2021). In addition, LINC00968 was found to be closely associated with ferroptosis (Lu et al., 2021) and N-6 methylation (m6A) (Zheng et al., 2021), and could attenuate drug resistance in cancer cells (Xiu et al., 2019). However, the prognostic value of five lncRNAs (RP11.1105O14.1, CTD.2555C10.3, RP11.462L8.1, AC018647.3, and CTD.3179P9.1) for cancer and their contribution to apoptosis have been lacking studies. Therefore, further studies are needed to explore the role of these lncRNAs in LUAD and apoptosis.

There are complex interactions between tumor cells and the tumor microenvironment that significantly influence tumor progression (Arneth, 2020). Therefore, this study demonstrates the relationship between ApoRLSig and tumor immune microenvironment. Significant differences in immune cell infiltration were found between high- and low-risk groups, confirming the role of ApoRLs in the regulation of tumor immune infiltration. Tumor immunity depends on the balance between immune cells that promote tumor or inhibit tumor progression (Wang et al., 2019). Type 1 T helper cells, which release TNF-a, IL-2, and interferon-g (IFN-g), exert antitumor effects, while Type 2 T helper cells mainly produce IL-4 to suppress the host immune system and promote tumor growth (Becker, 2006). M1 macrophages and natural killer cells have been shown to exert antitumor effects during tumorigenesis, and natural killer cells can drive tumor immunotherapeutic responses (Biswas et al., 2008; Huntington et al., 2020). It has been shown that the presence of CD8^+^ T cells is a hallmark of the anti-tumor immune response (Chen Y. et al., 2021a). Dendritic cells are specialized antigen-presenting cells that play a key role in the initiation, programming and regulation of tumor-specific immune responses (Melief, 2008; Li and He, 2018). CD4^+^ T regulatory cells, MDSC and mast cells may promote tumor progression (Ostrand-Rosenberg, 2008). In addition, an increase in neutrophil count is often strongly associated with poor cancer prognosis (Mollinedo, 2019). Consistent with previous studies, our study found more infiltration of immune cells performing anti-tumor responses (e.g., Activated CD8^+^ T cells, Type 1 T helper cells, Activated dendritic cells, M1 macrophages, and Natural killer cells) in the tumor microenvironment of patients with low risk scores, reflecting a reduction in malignancy and the effects of various treatments. In contrast, more immune cells that promote tumor progression (e.g., CD4^+^ T regulatory cells, mast cells, and neutrophils) were found in high-risk scoring patients. An exception emerged, with higher levels of MDSC infiltration in the low-risk population. Lung cancer has high levels of MDSCs, which are associated with resistance to chemotherapy, targeted therapy and immunotherapy and can predict poor prognosis (Liu et al., 2010; Feng et al., 2012; Heuvers et al., 2013; Huang et al., 2013; Zhou et al., 2018). This also explains the fact that patients with low risk scores are less sensitive to multiple chemotherapeutic agents than patients with high risk scores in our study.

In addition, apoptosis not only plays an important role in tumor development, but also has an impact on the effectiveness of immunotherapy and molecular targeted therapy for tumors (Carneiro and El-Deiry, 2020; Michie et al., 2020). Since immunotherapy with checkpoint inhibitors plays a key role in LUAD, we further investigated the differences in the expression of ICGs between high- and low-risk groups. The expression levels of HLA-DPB1, KIR2DL4, CD274, PVR, CD276, HLA-DRA, HLA-DOA, HLA-DRB5, HLA-DPA1, and HLA-DRB1 were found to be significantly different in the two groups of patients. Meanwhile, we found higher expression levels of most immune checkpoint genes in patients with high risk scores, prompting a superior immunotherapy effect in the high-risk group than in the low-risk group. Of note, patients with high risk scores were found to be highly sensitive to the chemotherapeutic agents cisplatin, docetaxel, gemcitabine and paclitaxel, indicating that the high-risk group was also outperformed by the low-risk group in terms of chemotherapy efficacy. These results suggest that lncRNAs in this signature may influence the development of LUAD by regulating immune responses in tumors and play a crucial role in chemotherapy drug resistance in LUAD.

The strength of this study is that we have constructed the first prognostic model of ApoRLs in LUAD and analyzed the relationship of the risk score with immunotherapy response and chemotherapy drug sensitivity. Most importantly, the lncRNA signature constructed in this study has higher predictive accuracy and is more comprehensively studied than another existing apoptosis-related signature that is used to predict the prognosis of lung adenocarcinoma (Zou et al., 2022). However, there are limitations in our study. First, we used only one dataset to construct the model. Second, this is a retrospective study. Third, this study lacks functional experimental validation. Hence, prospective cohort studies and molecular biology experiments are needed in this study to further validate the prognostic value of ApoRLSig and to explore the molecular mechanisms of ApoRLs.

In summary, we constructed a novel ApoRLSig to predict the prognosis of LUAD patients, and established an effective nomogram model including ApoRLSig. Furthermore, the most important contribution of this study is that we demonstrated the relationship between ApoRLSig and tumor immune microenvironment and further evaluated the relationship between ICGs, chemotherapy drug sensitivity, and risk score.These findings are of great importance in guiding the treatment and prognostic evaluation of patients with LUAD.

## Data Availability

RNA sequencing (RNA-seq) data and corresponding clinical survival information for TCGA-LUAD samples were downloaded through the UCSC xena website (https://xenabrowser.net/datapages/). Apoptosis-related genes can be found at hsa04210 in the KEGG pathway database (https://www.kegg.jp/kegg/pathway.html). c5.go.v7.5.1.entrez.gmt and c2.cp.kegg.v7.5.1.entrez.gmt were downloaded from the Molecular Signature Database (MSigDB; https://www.gsea-msigdb.org/gsea/msigdb/index.jsp).

## Abbreviations

ApoRLs, apoptosis-related lncRNAs; ApoRLSig, apoptosis-related lncRNA signature; AUC, the area under the curve; DE-ApoRLs, Differentially expressed ApoRLs; CGs, Immune checkpoint genes; KEGG, Kyoto Encyclopedia of Genes and Genomes; LASSO, the least absolute shrinkage and selection operator; lncRNAs, Long non-coding RNAs; LUAD, Lung adenocarcinoma; OS, overall survival; PCA, Principal component analysis; ROC, receiver operating characteristic; ssGSEA, the single sample gene set enrichment analysis; TCGA, The Cancer Genome Atlas
