# Supplementary material for: Identification of a apoptosis-related LncRNA signature to improve prognosis prediction and immunotherapy response in lung adenocarcinoma patients
Source: Front Genet. 2022 Sep 12;13:946939. doi: 10.3389/fgene.2022.946939 (PMC9510691; doi:10.3389/fgene.2022.946939)
Supplement: Supplementary file 1 [file Presentation1.zip › Suppl. Figure 1.DOCX]

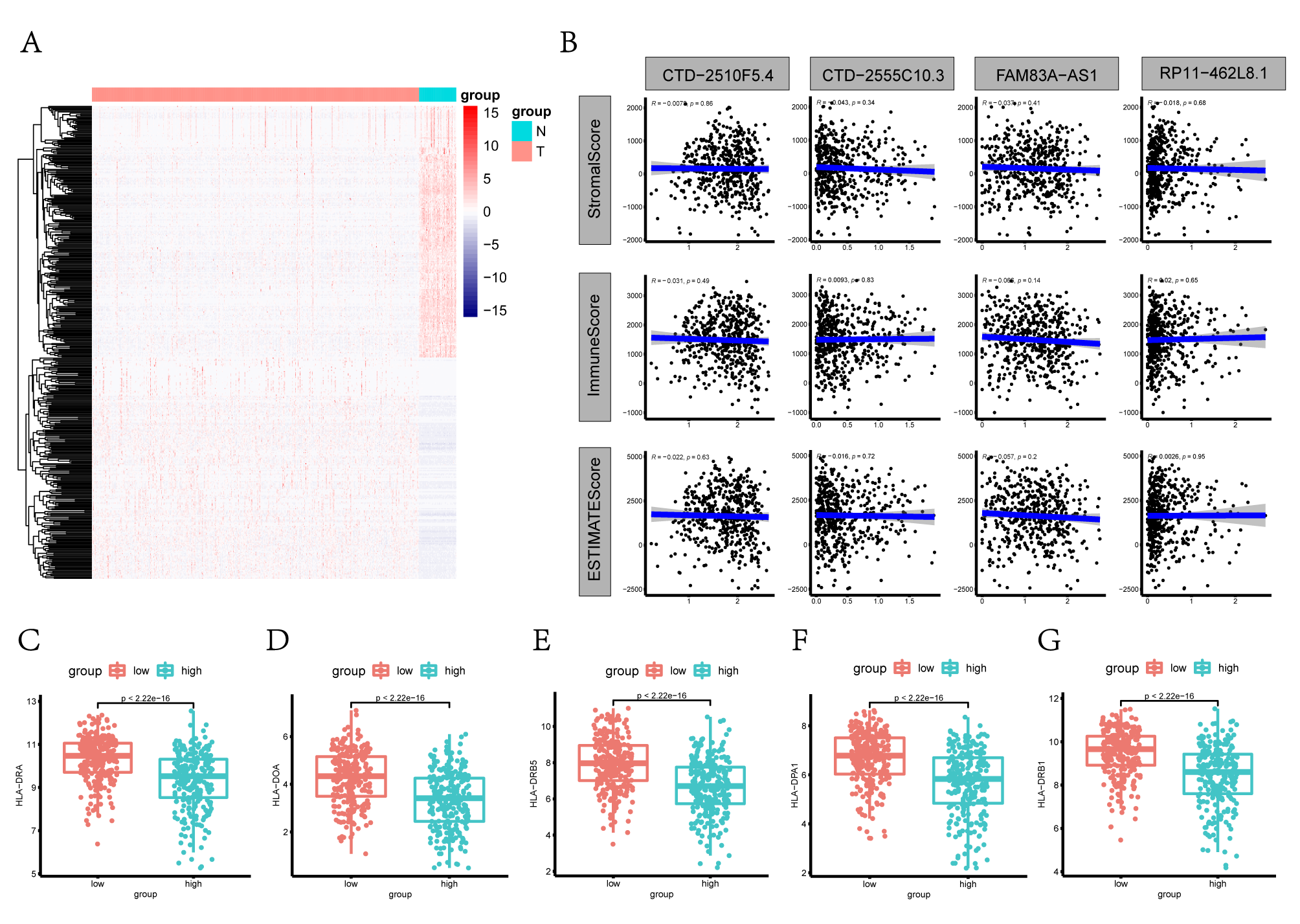


**Figure S1.** **Identification of prognostic apoptosis-related lncRNAs, and comparison of immune microenvironment and immune checkpoint genes between LUAD patients in the high-risk and low-risk groups. (A)** The differentially expressed apoptosis-related lncRNAs were shown in heatmap. (**B**) Correlation matrices between four lncRNAs expression and stromal score, immune score, and estimate score. The differential expression of five immune checkpoint genes, (**C**) HLA-DRA, (**D**) HLA-DOA, (**E**) HLA-DRB5, (**F**) HLA-DPA1, and (G) HLA-DRB1, between the high-risk group and the low-risk group.
